# Post-Mortem Appearances in Cholera Infantum, with Cases

**Published:** 1859-02

**Authors:** J. Lewis Smith

**Affiliations:** Late physician to the Northwestern Dispensary N. York.


*Post-mortem Appearances in Cholera Infantum, with Cases.* By J. LEWIS SMITH, M.D., late physician to the Northwestern Dispensary N. York.

SEVERAL months since, I communicated to the JOURNAL OF MEDICINE the records of eleven cases of cholera infantum, treated during the summer of 1857. Since then I have made the four following autopsies, and although they were necessarily not as thorough as I desired them to be, they throw additional light on the condition of the abdominal viscera in this fatal and much-dreaded disease.

The term cholera infantum is no doubt unwisely chosen to designate the complaint to which it is applied. The symptoms of the disease are not usually as rapid as we would infer from the name. During the summer months a large proportion of the children under two years of age in our large cities, have more or less relaxation of the bowels, and if this be at allsevere, especially if it be attended by constitutional disturbance, it receives the appellation of cholera-infantum. The symptoms of this disease may be summed up as follows: Frequent, thin acid evacuations, usually green, sometimes yellow or of a mixed color; irritability of stomach, sometimes commencing with the looseness, oftener at a later period; thirst; heat of surface, except in cases of prostration; accelerated pulse; natural respiration; no pain; no tenderness on pressure of the abdomen; progressive emaciation; sunken eyes; loss of strength. The complaint may terminate in recovery or death, in a few hours or days, or through many fluctuations after several weeks. It is evident that cases presenting the above symptoms may be termed by some diarrhœa, or enteritis, and I have thoght it proper to make the above remarks, that it may be known what I include in the expression, cholera-infantum. It will be seen that the lesions discovered last summer bore a general resemblance to those of the preceding, but differed from them in one or two respects.

*Case.* 1.—F., a female foundling, was placed, when a few days old, with a woman who ill-treated her, and gave her insufficient nourishment. On the third day of June last, when about two months old she was taken from this woman in an enfeebled condition, and was placed with a second nurse, in whose hands she died. After her second adoption she continued feeble, but without apparent disease, till the 28th day of June, when her bowels became relaxed.

On the 2d day of July the dejections became so frequent that the nurse took alarm, and brought her to the Dispensory for treatment.

At this time her eyes were sunken, limbs attenuated—the stools thin and greenish, and she vomited occasionally. Treatment, consisted mainly in the exhibition of laudanum, lime-water and brandy, availed little; she gradually failed, and died on the 12th day of July. She had no symptoms indicative of cerebral complication, and was apparently conscious till the last.

*Autopsy.*—Body emaciated, and eyes sunken; liver considerably enlarged, and of a more yellow cast than I had yet seen in this disease; stomach apparently healthy; red points and streaks on the mucous surface of the small intestines through their whole extent; the mucous membrane of the large intestines presented more disease than the jejunum and ileum, being red, thickened, and slightly ulcerated. These lesions existed in about an equal degree in the ascending and descending portions.

*Case* 2.—L., a male infant, was weaned on the first day of May, 1858, at the age of thirteen months and after this, his principal diet was milk from the country. His health continued good till about July 5, when his dejections became too frequent, and a few days subsequently, vomiting occurred. He was visited by an irregular practioner, who prescribed emetics daily, believing that the offending cause lay in the stomach. The vomiting and purging continued without any abatement, and he grew more and more emaciated till the 23d day of July, when I was called to see him.

He was now so emaciated that no reasonable hope could be indulged of his recovery. The pulse was accelerated, the tongue moist and slightly furred, the stools watery, of an acid reaction, and numbering from eight to twelve daily. By means of brandy and lime-water, the vomiting was arrested, but otherwise there was no improvement. The discharges continued thin and frequently, and the emaciation and debility slowly but progressively increased. Besides the lime-water and brandy, which were given every two hours, the former in teaspoonful doses, the latter in doses of twenty drops, I ordered one drop of laudanum to be given three or four times daily.

When I had visited him about a week, sprue began to appear in his mouth, and for two days his stools contained considerable blood. After this, the dejections had a dark or slate color, and continued with about the same frequency till within a day of his death. On the first day of August, symptoms were noticed indicative of cerebral complication; he grew drowsy, frequently stroked his face or forehead, and on the sixth and seventh of the month, he held his head rigidly back, adducted his thumbs, cried out at intervals as if in great pain, and finally passed into a comatose state, in which he died on the seventh.

*Autopsy* on the following day. Body much emaciated, the intestines examined externally presented no noteworthy appearance, and the mesenteric glands were not enlarged; liver of a lighter hue, and apparently somewhat larger than natural; there were red points and streaks on the mucous surface of the stomach, but otherwise this organ seemed healthy; the small intestines, which were opened in several places, presented the usual appearance. I neglected, however, to examine the ileum, near the ileo-cæcal valves; the mucous membrane of the large intestines was vascular and thickened through its whole extent, but in two places only were there traces of continuous inflammation, viz., in the ascending portion, and in the sigmoid, two lines in breadth, and apparently not reaching to the muscular coat. The other viscera were not examined.

*Case* 3.—On the 19th day of September 1858, I was asked to visit L. K., a female infant thirteen weeks old and in a dying condition. I was informed that the child's mother had died when she was a few weeks old, after which she had been nourished by the bottle, and that she had been with her present nurse only two weeks. At the time of her adoption by this nurse, her stools numbered five or six in twenty-four hours, but the discharges became more frequent, and during the four days preceding my visit had reached the number of ten or twelve daily. She also had attacks of vomiting and was feverish. Three days before her death, symptoms of cerebral congestion or effusion occurred; the thumbs were adducted, and drowsiness and finally spasms supervened. She died a few hours after my visit in a comatose state.

*Autopsy.*—Body somewhat emaciated; the stomach and intestines examined externally presented no noteworthy appearance; stomach was moderately distended with a liquid resembling water and coagulated milk, in which were dark streaks, probably of blood; the mucous membrane of the stomach did not appear to have undergone any alteration, having the light pinkish hue of infancy; in the small intestines there was a similar absence of any lesion except near the ileo-cæcal valves, where the mucous-membrane was somewhat thickened, and the Peyerian patches unusually distinct; the contents of the stomach and small intestines gave a slightly acid reaction.

In the large intestines the mucous membrane, from the ileo-cæcal valves to the rectum, was mottled with red points and streaks, and thickened; these lesions were found in about equal intensity in all parts of the colon; with the back of the scalpel, bloody mucus could be scraped from the surface of the colon, and this surface contained either small ulcerations or hypertrophied mucous follicles. The liver had apparently its usual color, size and consistence.

*Case* 4.—R., a nursing female infant, was taken on the 28th day of July, 1858, when about six weeks old, with looseness of the bowels. The mother was healthy and there was no obvious cause of the attack except the high temperature of the season. A homœopathic physician attended her till about the 16th day of July, when he was discharged, as the symptoms were daily becoming more alarming. At this time she was rather emaciated. she had a cough from a moderate bronchitis; her pulse was somewhat accelerated; her stools, which were thin and green, numbered six to twelve daily and gave an acid reaction; she vomited occasionally, continued to nurse and was very fretful. I prescribed aquæ calcis ʒj. every two hours if the discharges continued acid: tinc. opii gtt. ¼ according to the frequency of the evacuations, and wine or brandy.

From this date till the 29th day of August the looseness continued with but few intermissions, though considerably checked by the medicines. On the 29th, as there was no new symptom, my visits were discontinued. On the 19th of September, just three weeks after my last visit, I was called again to see the child and fonnd her dying. The limbs were cold, eyes bleared, and the respiration short and at long intervals. She could not be aroused and was much emaciated though her passages, the parents stated, had been nearly healthy during the past few days. Death occurred soon after.

*Autopsy.*—Body emaciated and eyes sunken; the mucous membrane of the stomach presented its usual appearance. The mucous membrane of the small and the large intestines, thoroughout their whole extent was vascular and thickened in places; the fecal matter in the intestines had a muco-sanguineous appearance, which is more noteworthy as the last dejections seemed healthy. With the back of the knife I could scrape blood with the mucus from the mucous membrane. The jejunum and ileum were as much diseased as the colon, and in no part of the intestines were ulcerations noticed. The liver and kidneys were apparently healthy; the spleen had a leaden color.

The following is a resume of the post-mortem appearances narrated above:

*Stomach.*—In three cases this organ seemed healthy; in the remaining case it was vascular, a condition due perhaps to brandy, as the child took this stimulant at intervals of two to four hours, during the last two weeks of his life. In a paper on Cholera Infantum published by me in the July number of the Journal, and which contained my observations for the year 1857, it was stated that the stomach contained no appreciable lesion in five cases which I had examined, so that in eight out of nine of my post-mortem examinations, in cases of cholera infantum, this viscus has had a healthy appearance.

*Small intestines.*—In two of that four cases the small intestines were more or less inflamed throughout their whole extent; in one they seemed healthy except near the valves, and in the other case thejejunum and the upper part of the ileum were also healthy, the part near the colon not being examined. These cases, so far as they go, agree with those published in my former paper, in the fact that the small intestines were not uniformly inflamed, and that the ileum may be affected when the jejunum escapes.

*Colon.*—In all four cases this was inflamed, both the ascending transverse, and the descending portions. In three of these the inflammation seemed pretty uniform; in the other the point of most intense inflammation was the sigmoid flexure. In three cases the records state that the mucous membrane of the colon was thickened; in the examination of the other patient no note was made of this fact. In two of the autopsies the colon was found distinctly ulcerated. and in one of the other two, there were either slight ulcerations or else unusual distinctness of the mucous follicles, while in the remaining case no ulceration was present.

In the above cases, then, as well as in the eleven cases whose histories are given in the July number, the colon was without exception the seat of inflammation; but these cases differed from those in the fact that the ascending portion, with one exception, was as much diseased as the descending.

*Liver.*—In two of the examinations this organ contained no lesion appreciable to the naked eye; in the other two it presented a lighter hue than the improverished condition of the blood would be likely to cause, and in one these last there was considerable enlargement. Dr. Gouley, whose zeal and accuracy as a microscopist are well known in this city, has lately informed me that in several instances he has found considerable fatty deposition in the liver in cases of cholera infantum. I am now induced to think the morbid condition mentioned by him, may have been present not only in the above cases in which the liver had the color characteristic of fatty disease, but in some of the cases examined by me in 1857, as the naked eye cannot well discover this lesion unless the amount of fat be considerable.

It is worthy of remark that in all the post-mortem examinations made by me in cases of cholera infantum, now fifteen, there have been evident traces of inflammation in the colon, usually of a severe grade. Those who believe that the disease is not essentially inflammatory, can find an explanation of this in the fact that the evacuations are so acid. During the past two summers I have carried litmus paper with me in my visits to children affected with this complaint; and in all cases, except in the advanced stages of the disease, when there was reason to think ulceration was present, have found the dejections acid. What the relation of this acidity is to the inflammation, whether as cause or effect, is not easily determined. The fact that an erythematous blush, or even excoriation, so soon occurs around the anus in cholera infantum tends to show that the stools are sufficiently irritative to cause intestinal inflammation. On the other hand, some excellent authorities in infantile pathology state that the evacuations are commonly acid in the entero-colitis of chidren. I have formed no opinion as to which has the precedence, the acidity or the inflammation; but I have known the stools to be acid at the very commencement of the complaint.

Another point of interest in reference to the above cases is the fact that three of the four children had not yet reached the age of dentition. As there has been thought to be an intimate relation between the summer complaint of children and dentition, the following table of one hundred and forty-three cases, prepared from my note-book and showing the stage of this process, will be interesting:

| | |
|---|---|
| Have no teeth | 25 |
| Cutting incisors | 54 |
| Have incisors, cutting first molars | 17 |
| Have incisors, first molars, cutting canine teeth | 22 |
| Have incisors, first molars. canine teeth, cutting last molars | 7 |
| Have all the teeth | 15 |
| Total | 143 |

From this it appears that the disease is most frequent during the first half of dentition, and is comparatively rare when the broad surfaces of the second or last molars are cutting the gum. It is interesting also to observe that the disease does occur before and after dental evolution. Both these facts go to show that the influence of teething in the production of cholera infantum must be subordinate.

Tee following table will show more accurately the age at which the complaint usually takes place:

| AGE | NUMBER |
|---|---|
| Five months and under | 33 |
| From 5 to 12 months | 857 |
| " 12 " 18 " | I24 |
| " 18 " 24 " | 14 |
| " 24 " 30 " | 9 |
| " 30 " 36 " | 2 |
| Over 36 months | 9 |
| Total | 398 |

This table shows that the summer complaint of children is rare before the fifth month, and after the second year, so as to corresponid with the time of increase in the size of the mucous follicles as it is given by Billard and others.—*The New York Journal of Medicine*.

